# Commissuroplasty for the anterior commissure defect caused by tricuspid valve endocarditis using patch closure and modified placement of a rigid ring

**DOI:** 10.1186/1749-8090-9-36

**Published:** 2014-02-20

**Authors:** Jong Hun Kim, Kyung Hwa Kim, Jong Bum Choi, Ja Hong Kuh

**Affiliations:** 1 Department of Thoracic and Cardiovascular Surgery and Research Institute of Clinical Medicine, Chonbuk National University Medical School, 20 Geonji-Ro, Deokjin-Gu, Jeonju, Chonbuk 561-712, Republic of Korea

**Keywords:** Heart, Valve, Tricuspid valve, Endocarditis, Tricuspid valve repair

## Abstract

The anterior commissure defect of tricuspid valve may leave remnant regurgitation with inadequate leaflet coaptation after simple patch closure of the defect. A 19-year-old man underwent commissuroplasty for the anterior commissure defect caused by tricuspid valve endocarditis. Significant remnant regurgitation after patch closure of the anterior commissure defect was successfully solved by modified placement of an undersized rigid ring along the functional valve opening. The anterior horn of the rigid ring was affixed to the medial margin of the patch to reinforce the new commissure and enhance leaflet coaptation.

## Background

Tricuspid valve endocarditis is very rare in young healthy individuals with no history of injection drug use. The abscess-type endocarditis without vegetation accounts for only 1.6% of all cases of native valve endocarditis [1]. A young healthy man without a history of drug addiction was found to have right-sided abscess-type endocarditis. He presented with severe tricuspid valve regurgitation associated with disruption of the medial papillary muscle of the tricuspid valve. We report a case of commissuroplasty for the anterior commissure defect due to tricuspid valve endocarditis, which consisted of patch closure of the commissure defect and modified placement of a rigid ring for complete leaflet coaptation.

## Case presentation

**Figure 1:**
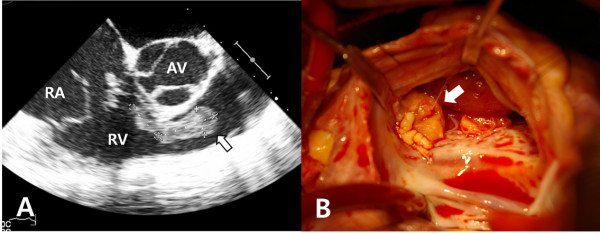
**Preoperative transesophageal echocardiogram and gross operative findings. A**. The tricuspid valve had prolapsed leaflets with coaptation failure and a mobile mass of 2.4 cm × 0.8 cm (white arrow) attached to the ventricular septum under the valve. **B**. An abscess lump (white arrow) was attached to ventricular septum; it was just at the base of the disrupted medial papillary muscle. (AV = aortic valve; RA = right atrium; RV = right ventricle).

**Figure 2:**
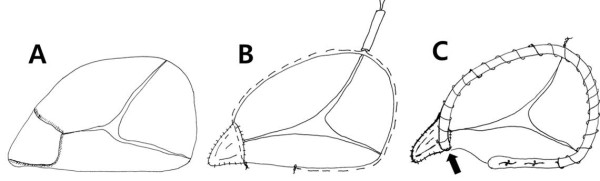
**Operative procedures. A**. After the prolapsed leaflet segments and chordae were excised, the anterior commissural defect was made. **B**. The defect was closed with an elliptical pericardial patch of 2.0 × 1.0 cm size. An adjustable DeVega-type annuloplasty using two continuous 5–0 Polypropylene sutures was performed to select an appropriate-size ring for complete leaflet coaptation. **C**. A 26-mm Edward MC^3^ ring was placed using two interrupted, pledgeted 2–0 Dacron sutures and two continuous 3–0 polypropylene sutures. The anterior horn of the rigid ring (black arrow) was sutured to the medial end of the patch.

**Figure 3:**
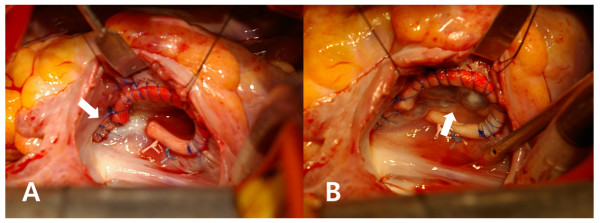
**Operative findings. A**. The anterior commissure defect was closed with a patch (white arrow) and a rigid ring was placed along the functional valve opening. **B**. The valve leaflets showed complete coaptation (white arrow) on saline test.

A 19-year-old Korean man without any medical history was admitted to the authors’ institution with complaints of high fever greater than 39.0°C, chill, and myalgia for one week. Transthoracic echocardiography at that time revealed severe tricuspid regurgitation, prolapse of the leaflets, and a mobile mass just distal to the tricuspid valve. *Staphylococcus aureus* was grown in blood cultures. His medical history prior to admission was only significant for repeated purulent infections in his right ingrown toenail for three months. Chest CT scans on admission demonstrated acute multifocal bronchopneumonia in both lungs. Transesophageal echocardiography showed prolapsed valve leaflets with coaptation failure and a mobile mass of 2.4 cm × 0.8 cm size between the tricuspid valve and the right ventricular outflow tract (Figure 1A). On the admission day, the ingrown toenail was treated by partial avulsion medially. *Staphylococcus aureus* was also cultured from the toenail discharge. On the third hospital day, surgery was done under moderate hypothermic cardiopulmonary bypass. The medial papillary muscle supporting the anterior commissure of the tricuspid valve was completely disrupted from the ventricular septum, to which a 2.0-cm abscess lump covered with a thin, friable and smooth membrane was attached (Figure 1B). The abscess was readily aspirated by a general sucker and the abscess base was cleaned with a curette. The disrupted papillary muscle and chordae and the inflammatory leaflet segment in the anterior commissure were excised (Figure 2A). The commissural defect was augmented with an elliptical glutaraldehyde-treated autologous pericardial patch of 2.0 cm × 1.0 cm size using a continuous 5–0 polypropylene suture (Figure 2B). The valve remained incompetent on saline test. A 28-mm MC^3^ annuloplasty ring (Edwards Lifesciences LLC; Irvine, CA, USA), which was chosen by the anterior leaflet area, was placed along the anatomical annulus, but the valve was still incompetent. After the ring was removed, a type of DeVega annuloplasty using a single row of 5–0 polypropylene sutures was performed. The annular plication suture was tightened until complete leaflet coaptation without regurgitation was achieved on saline test under occlusion of the main pulmonary artery (Figure 2B) [2]. The complete leaflet coaptation occurred when the annular size was reduced to a No. 26 ring sizer (Model 1175 Sizers; Edwards Lifesciences LLC). A 26-mm MC^3^ ring was placed using two interrupted mattress 2–0 Dacron sutures near the atrioventricular node and two continuous 3–0 polypropylene sutures (Figure 2; Figure 3A). The anterior horn of the ring was sutured to the medial end of the attached patch instead of the anatomical annulus so that it could be placed along the margin of the functional valve opening. The valve showed a good coaptation on saline test (Figure 3B). Early postoperative echocardiograms showed good valve competence. Follow-up echocardiography at 16 months postoperatively revealed trivial regurgitation, with a peak velocity of 1.3 m/sec and a mean transvalvular gradient of 3.0 mmHg. With peak treadmill exercise (stage IV, 13.3 METS by Bruce protocol), the transvalvular peak velocity and mean gradient were 2.4 m/sec and 8.0 mmHg, respectively.

## Discussion

In the existing literature on valve repair of infected atrioventricular valve endocarditis [3,4], cusp commissuroplasty consisted of resection of the infected cusp, a single stitch to form a coaptation zone, and patch closure of the cusp defect. In our case, however, the new coaptation zone of the anterior commissure has a burden of tension and is supported by the adjacent thin and weak chordae of the anterior and septal leaflets. The concerning problems can be settled by placement of the anterior horn of the rigid ring on the new coaptation zone. In addition, because the ring was placed along the closure margin of the functional valve opening, an effective annular plication for leaflet coaptation could be performed.

To select an adequate ring size for complete leaflet coaptation and valve competence, adjustable annuloplasty was performed using continuous 5–0 polypropylene annular sutures and an undersized rigid ring [2]. On follow-up echocardiography, the young man with a body surface area of 1.7 m^2^ showed insignificant peak and mean valve gradients at peak exercise as well as at rest, suggesting that the ring size was likely appropriate for the patient [5]. The mean valve gradient at rest was lower than in cases of tricuspid valve replacements [6].

## Conclusion

In cases of anterior commissure defect due to tricuspid valve endocarditis, placement of the anterior horn of a rigid ring on the new coaptation zone (new commissure) after patch closure of the defect can reinforce the new commissure and ensure effective annular plication for complete leaflet coaptation. This technique may be useful and reproducible, especially in patients with tricuspid valve regurgitation associated with anterior commissure defect.

## Consent

Written informed consent was obtained from the patient and his parents for publication of this case report and accompanying images. A copy of the written consent is available for review by the Editor-in-Chief of this journal.

## Abbreviations

AV: Aortic valve; CT: Computed tomography; RA: Right atrium; RV: Right ventricle.

## Competing interests

The authors declare that they have no competing interests.

## Authors’ contributions

JHK and KHK wrote the draft of the manuscript and obtained the written consent. JBC and JHK performed the literature review, participated in manuscript writing, and gave final approval of the manuscript. All authors have read and approved the final manuscript.
